# The prevalence and molecular mechanisms of mupirocin resistance in *Staphylococcus aureus* isolates from a Hospital in Cape Town, South Africa

**DOI:** 10.1186/s13756-020-00707-8

**Published:** 2020-03-14

**Authors:** Shima M. Abdulgader, Tshepiso Lentswe, Andrew Whitelaw, Mae Newton-Foot

**Affiliations:** 1 Division of Medical Microbiology, Faculty of Medicine and Health Sciences, Stellenbosch University and NHLS, Tygerberg Hospital, Francie van Zijl Drive, PO Box 241; Cape Town, Tygerberg, 8000 South Africa; 2 National Health Laboratory Service, Tygerberg Hospital, Cape Town, South Africa

**Keywords:** *Staphylococcus aureus*, MRSA, Mupirocin, *spa-*typing, Antimicrobial resistance, South Africa

## Abstract

**Background:**

Antimicrobial resistance is an increasingly serious problem in public health globally. Monitoring resistance levels within healthcare and community settings is critical to combat its ongoing increase. This study aimed to describe the rates and molecular mechanisms of mupirocin resistance in clinical *Staphylococcus aureus* isolates from Tygerberg Hospital, and to describe its association with strain types.

**Methods:**

We retrospectively selected 212 *S. aureus* isolates which were identified from blood samples and pus swabs during the years 2009–2011 and 2015–2017. The isolates were identified using conventional microbiological methods and genotyping was done using *spa* typing. Cefoxitin (30 μg) disc diffusion and the two disc strategy (5 μg and 200 μg) were used to determine susceptibility to methicillin and mupirocin, respectively. Isolates with high-level resistance were screened for the plasmid mediated genes *mupA* and *mupB* by PCR, and sequencing of the *ile*S gene was done for all isolates exhibiting low-level resistance to describe the mutations associated with this phenotype. Chi-square test was used to assess the associations between mupirocin resistance and *S. aureus* genotypes.

**Results:**

Of 212 *S. aureus* isolates, 12% (*n* = 25) were resistant to mupirocin, and 44% (*n* = 93) were methicillin resistant. Strain typing identified 73 *spa* types with *spa* t045 being the most predominant constituting 11% of the isolates. High-level mupirocin resistance was observed in 2% (*n* = 5), and low-level resistance in 9% (*n* = 20) of the isolates. The prevalence of high-level mupirocin resistance amongst MRSA and MSSA was 4 and 1% respectively, while the prevalence of low-level mupirocin resistance was significantly higher in MRSA (18%) compared to MSSA (3%), (*p* = 0.032). *mupA* was the only resistance determinant for high-level resistance, and the *IleS* mutation V588F was identified in 95% of the isolates which showed low-level resistance. A significant association was observed between *spa* type t032 and high-level mupirocin resistance, and types t037 and t012 and low-level resistance (*p* <  0.0001).

**Conclusion:**

The study reported higher rates of low-level mupirocin resistance compared to high-level resistance, and in our setting, mupirocin resistance was driven by certain genotypes. Our study advocates for the continuous screening for mupirocin resistance in *S. aureus* in clinical settings to better guide treatment and prescribing practices.

## Introduction

*Staphylococcus aureus* is the second most frequent cause of nosocomial bloodstream infections worldwide [1]. *S. aureus* nasal carriage is a risk factor for subsequent infections, especially amongst surgical and dialysis patients [2, 3], and carriage of methicillin-resistant *S. aureus* (MRSA) is a particular problem in these high risk patients due to the limited therapeutic drugs available to treat post-operative infections [4]. Consequently, infection prevention strategies such as nasal decolonization are employed to minimize the occurrence of staphylococcal infection and reduce the risk of transmission within healthcare settings [5, 6]. In routine intensive care unit practice, universal decolonization has proven more effective in reducing nosocomial bloodstream infections caused by MRSA as well as any other pathogen, compared to a targeted approach [7]. In some settings, routine screening and decolonisation of MRSA carriers prior to hospital admission is also practiced [8]. The intranasal application of the antibiotic mupirocin (2%) 2–4 times daily for 4–7 days is an effective and affordable strategy for decolonization of MRSA, used alone or in combination with 4% chlorhexidine gluconate (CHG) based body wash [9, 10]. Mupirocin, also known as pseudomonic acid A, is naturally produced by *Pseudomonas fluorescens,* was first isolated in 1971 and first introduced into clinical practice in the United Kingdom in 1985 [5, 11]. It is also used as a topical agent to treat localised skin and soft tissue infections [10]. Mupirocin inhibits protein synthesis by binding to the bacterial isoleucyl-tRNA synthetase enzyme which is encoded by the *ile*S gene [11].

Resistance to mupirocin in *S. aureus* emerged largely due to long-term, unrestricted and unjustified use [12, 13]. Mupirocin resistance is phenotypically categorized into two levels based on the minimum inhibitory concentration (MIC); low-level resistance with MICs of 8–256 mg/ml, and high-level resistance with MICs > 512 mg/ml [4, 14]. The molecular mechanism of low-level mupirocin resistance involves point mutations in the *ile*S gene; V588F and V631F are two common mutations associated with this phenotype [15–17]. High level mupirocin resistance is mediated by the plasmid encoded genes *mup*A and *mup*B, which encode an alternative isoleucyl-tRNA synthetase (*ile*S2) that is not targeted by mupirocin [18, 19].

Mupirocin resistance in *S. aureus*, especially high-level resistance, is a serious clinical problem, since it is associated with failure of decolonization, especially among MRSA carriers [20]. This is of particular concern for infection prevention and control practices involved in the management of MRSA outbreaks and in the pre-admission management of surgical patients to minimize post-operative MRSA infections [10]. A recent systematic review describing mupirocin resistance in Africa highlighted the scarcity of data and advocated for the need for surveillance studies to monitor the levels of mupirocin resistance both in the community and healthcare settings [6]. Screening for mupirocin resistance has only been carried out in 12 of the 54 African countries, with an estimated pooled prevalence of 14% resistance amongst MRSA isolates. Only six studies have been conducted in South Africa reporting prevalences of 7–50%, 4–47%, and 0.9–23%, for overall mupirocin resistance, low-level and high-level mupirocin resistance, respectively, in MRSA [6]. There is a paucity of data describing associations between mupirocin resistance and the genetic background of *S. aureus* in Africa. However, studies from the United States as well as low-middle income countries such as Iran have reported associations between *spa* types t002, t008, and t064 and mupirocin resistance [21, 22]. Few studies have described mupirocin resistance in the public healthcare sector in the Western Cape of South Africa, however, they reported aggregated data from multiple provinces across South Africa, and mainly investigated resistance rates in MRSA isolates [23–25]. Therefore this study aimed to describe the rates of mupirocin resistance in *S. aureus* isolates collected at Tygerberg Hospital, to describe the associations between mupirocin resistance and the genotypes which are circulating in our setting, and to investigate the molecular mechanisms of low- and high-level mupirocin resistance.

## Methods

This study took place at Tygerberg Hospital, a 1384 bed tertiary academic hospital that serves a population of approximately 1.9 million in the Western Cape, South Africa. *S. aureus* isolates were identified at the National Health Laboratory Service (NHLS) microbiology laboratory as part of the diagnostic investigation of patients presenting to the hospital. Our Biobank was retrospectively searched for any *S. aureus* isolates which had been collected from clinical specimens between the years 2009 and 2017. A subset of these isolates was randomly selected to be included in this study. Information on the date of collection and the type of clinical specimen were retrieved from laboratory records. The study was approved by the Health Research Ethics Committee of Stellenbosch University (Reference number N14/06/065).

### Bacterial identification and strain typing

The selected *S. aureus* isolates were identified by standard microbiological methods such as Gram morphology, catalase, mannitol fermentation and DNase activity, and methicillin susceptibility was determined using cefoxitin disc diffusion, according to the CLSI guidelines [26]. Confirmed *S. aureus* isolates were stored at − 80 °C. All *S. aureus* isolates were genotyped using *spa* typing as described previously [27].

### Mupirocin susceptibility testing

The stored isolates were streaked on Tryptone Blood Agar (Diagnostic Media Products, South Africa). Overnight cultures were used to perform mupirocin susceptibility testing on Muller-Hinton Sensitivity medium using two different mupirocin containing discs: a 5 μg disc to detect low-level mupirocin resistance and a 200 μg disc to detect high-level mupirocin resistance (Mast diagnostic group, United Kingdom). Zones of inhibition were interpreted according to the CLSI guidelines [26].

### Molecular mechanisms of mupirocin resistance

*S. aureus* isolates which exhibited high-level mupirocin resistance were screened for the presence of the plasmid mediated *mup*A and *mup*B genes using the primers and conditions published previously [28]. For low-level resistance, a 450 bp region in the *ile*S gene was amplified using the modified primers *mup*LL-F1 5’CCGGAATTAAGTTTCCCAGC-3′ and *mup*LL-R 5′ CAAAGTTTTCATAGTTGTTAATCGT3’ [29]. Sanger sequencing was done to describe the presence of point mutations within the *ile*S gene.

### Statistical analysis

Statistical analysis was done using STATA version 12 (StataCorp LLC, USA). The difference in the rates of mupirocin resistance between MRSA and MSSA was determined using z-test. Chi-square test was used to assess the associations between strain type and mupirocin resistance. Statistical significance was defined as *p*-values of < 0.05.

## Results

### Rates of mupirocin resistance

We included 212 *S. aureus* isolates based on the availability in the Biobank. Of these, 93 were collected between 2009 and 2011, and 119 between 2015 and 2017. Ninety-two percent (*n* = 194) of the isolates were from blood cultures and 8% (*n* = 18) were isolated from pus swabs. Forty four percent (*n* = 93) of the isolates were MRSA. Strain typing was successful for 180 isolates and 73 *spa* types were identified.

**Fig. 1:**
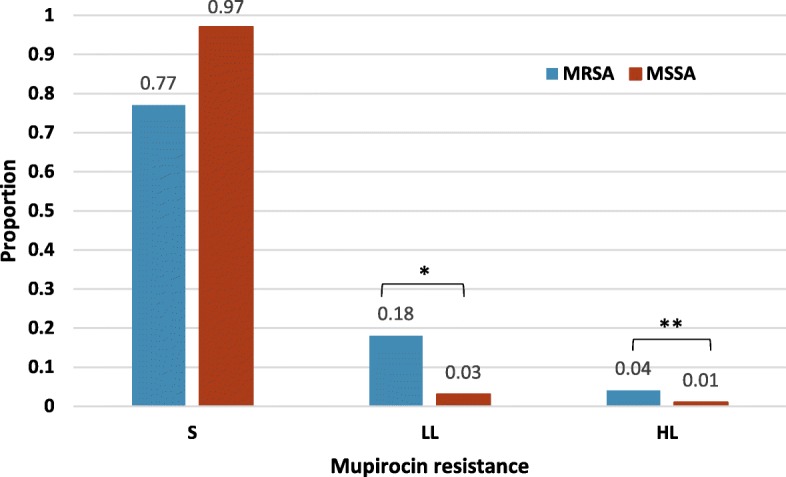
Distribution of mupirocin resistance among *S. aureus* isolates categorized by methicillin-susceptibility. S: mupirocin susceptible; LL: Low-level mupirocin resistance; HL: high-level mupirocin resistance. The asterisks show the significant differences in the resistance rates between MSSA and MRSA. * *p*-value =0.032, ** *p*-value < 0.005

Mupirocin resistance was observed in 12% (*n* = 25) of the isolates; five (2%) and 20 (9%) exhibited high-level and low-level resistance, respectively. The prevalence of mupirocin resistance was significantly different between MRSA (23%; *n* = 21), and MSSA (3%; *n* = 4) isolates (*p* = 0.04) (Fig. 1).

This was consistent for low- and high-level mupirocin resistance; with low level resistance rates of 18% (*n* = 17) among MRSA and 3% (*n* = 3) among MSSA (*p* = 0.032), and high-level mupirocin resistance rates of 4% (*n* = 4) and 1% (*n* = 1) among MRSA and MSSA, respectively (*p* <  0.005).

### Correlation between mupirocin resistance and strain types

**Fig. 2:**
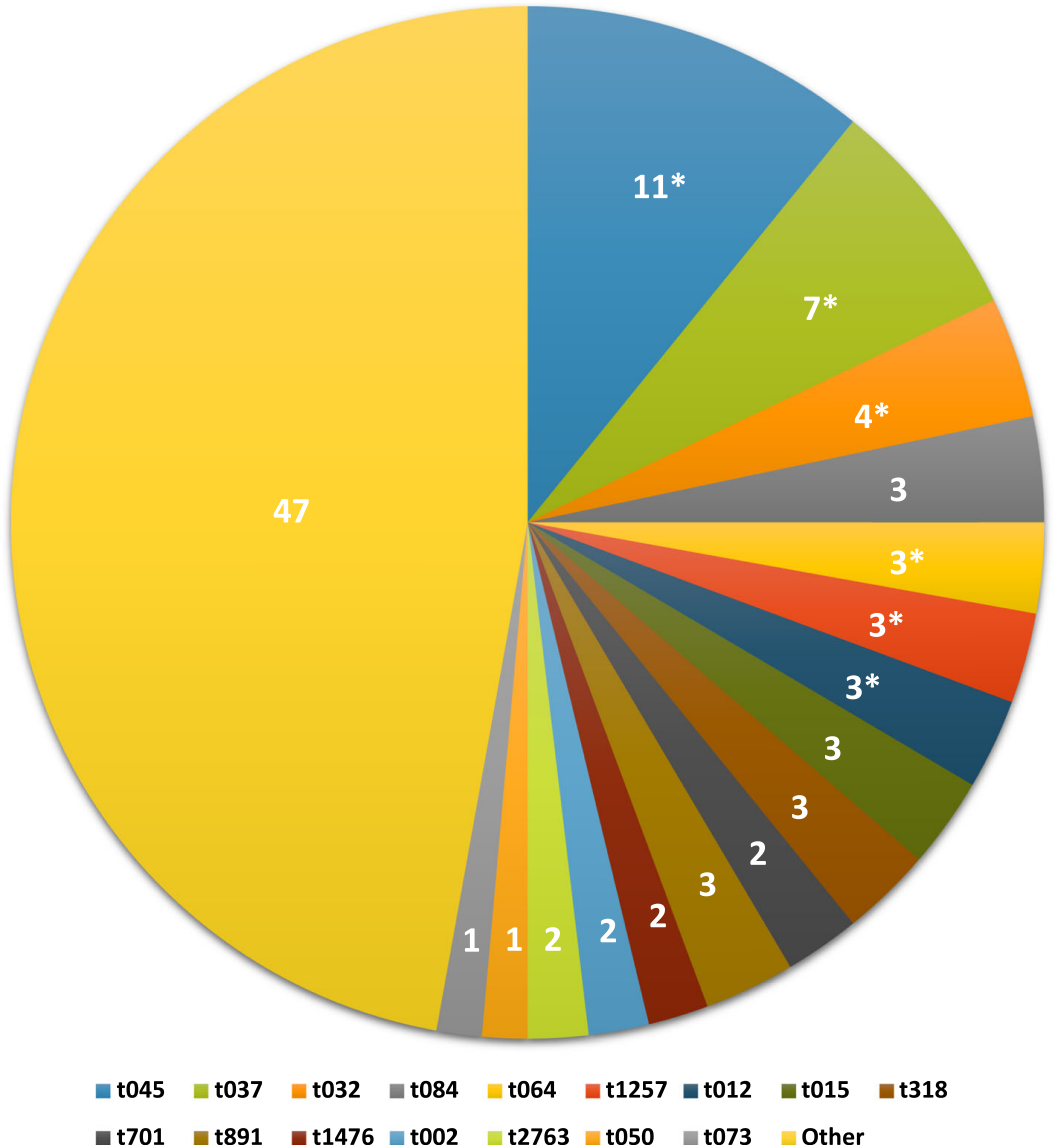
Distribution of the *spa* types identified in all the clinical *S. aureus* isolates. The data shown are the percentages of the different *spa* types. The asterisks indicate the *spa* types that contained only MRSA isolates. “Others” represent the *spa* types with ≤2 isolates

Figure 2 shows the *spa* type distribution in the isolate collection, with *spa* types identified in ≤2 isolates (*n* = 100) grouped as “others”. Type t045 was the most common, representing 11% of isolates, followed by t037 which constituted 7% of the isolate collection. Of the 16 most common *spa* types (*n* = 112), six contained only MRSA isolates (Fig. 2). Mupirocin resistance was represented in nine different *spa* types (Table 1). A significant association was noted between *spa* types t012 and t037 and low-level mupirocin resistance, while the *spa* type t032 was associated with high-level mupirocin resistance (*p* <  0.0001). Although all the isolates with *spa* type t045 were MRSA, they were all mupirocin susceptible. Seventy six percent of susceptible isolates belonged to *spa* types in which no resistant isolates were detected (Table 1).

**Table 1:** Correlation between *S. aureus* genotypes and mupirocin resistance

***Spa*** type	High-level N (%)	Low-level N (%)	Susceptible N (%)	Molecular mechanism	Methicillin resistance	***P***-value
**t037**	0 (0)	11 (55)	5 (3)	V588F	MRSA	< 0.0001
**t012**	0 (0)	5 (25)	1 (0.5)	V588F	MRSA	< 0.0001
**t032**	3 (60)	0 (0)	5 (3)	*mup*A	MRSA	< 0.0001
**t127**	1 (20)	0 (0)	0 (0)	*mup*A	MSSA	–
**t1467**	1 (20)	0 (0)	0 (0)	*mup*A	MRSA	–
**t891**	0 (0)	1 (5)	5 (3)	V588F	MSSA	–
**t1517**	0 (0)	1 (5)	1 (0.5)	V588F	MSSA	–
**t2360**	0 (0)	1 (5)	1 (0.5)	V588F	MSSA	–
**t073**	0 (0)	1 (5)	2 (1)	S570A	MSSA & MRSA	–
**Mupirocin susceptible*spa*types**	0 (0)	0 (0)	167 (88.5)	–	MSSA & MRSA	–
**Total**	5 (100)	20 (100)	187 (100)	–	–	–

Only significant *p*-values (Chi-square test) are shown in the table.

### Molecular mechanisms of mupirocin resistance

The *mupA* gene was detected in all five high-level mupirocin resistant isolates and the amplification product confirmed by Sanger sequencing. *mupB* was not detected, however the absence could not be confirmed as a positive control was not available. The absence of *mup*A and *mup*B was also noted in all isolates with low-level mupirocin resistance. The IleS V588F mutation was detected in 95% (*n* = 19) of the isolates with low-level resistance. One isolate (*spa* type t073) carried a possible novel *ile*S mutation, S570A.

## Discussion

Monitoring the levels of antimicrobial resistance within healthcare settings is critical to combat the ongoing increase in resistance. In the current study we sought to describe the rates and molecular mechanisms of mupirocin resistance at Tygerberg Hospital, and to determine possible associations between mupirocin resistance and specific *S. aureus* lineages.

Twelve percent of the isolates selected for this study exhibited resistance to mupirocin, and the prevalence of mupirocin resistance was significantly higher in MRSA (23%) than MSSA (3%). This trend is consistent with other studies across South Africa; 17% mupirocin resistance was described in MRSA and 2% in MSSA isolates collected from 13 public academic healthcare centres in Gauteng, KZN, Free-State and Western Cape in the years 2010–2012 [24]. The association between MRSA and resistance to a wide range of other antibiotics is well-established [30], and is considered a serious problem due to the limited available therapeutic drugs. In keeping with this, a study from the private sector in the Western Cape of South Africa, which investigated *S. aureus* isolates from all clinical specimens, reported 28% mupirocin resistance and a strong association between methicillin resistance and mupirocin resistance, and worryingly, co-resistance to mupirocin and fusidic acid (also a topical antibiotic used for skin infections) [30]. This highlights the need for stewardship of topical antimicrobial agents. The authors also recommended that in private healthcare sectors, antibiotic susceptibility testing should be performed before prescribing mupirocin to patients. We could not correlate the level of resistance with the use of mupirocin in our setting due to the limited access to this information. In our setting, mupirocin is used primarily for decolonisation as part of the management of MRSA outbreaks, although it is also sporadically used as a therapeutic agent for minor skin infections. This practice is under review as part of the hospital’s antimicrobial stewardship programme.

We reported only 2% high-level resistance as opposed to 23% in private healthcare sectors in the Western Cape, which suggests higher use of mupirocin in the private healthcare sector. Conversely, low-level resistance was 9% in our study compared to 4% in private healthcare sectors [30]. It is worth noting that more than 90% of the isolates in our study were from blood cultures compared to only 0.2% in the study from the private sector, where 77% of the isolates were from skin swabs. Evidence suggest that although invasive and nosocomial isolates (such as blood cultures) may be associated with a denser antimicrobial history, their mupirocin resistance rates are lower [23, 30, 31]. Of note, in previous studies, mupirocin resistance was highly associated within certain clinical practices such as plastic surgery, dermatology, and general medicine [30, 31]. Unfortunately, due to the retrospective nature of our study, we were unable to correlate our isolates with any clinical practices. This, as well as the issue of the overrepresentation of blood culture isolates should be addressed in future studies.

Clinically, high-level mupirocin resistance is associated with decolonization failure, however, recent studies have shown that low-level resistance is also associated with reduced effectiveness of mupirocin in eradicating MRSA carriage [17, 32, 33]. We detected higher rates of low-level mupirocin resistance than high-level resistance. The *mup*A gene was the only resistance determinant observed in the five high-level resistant isolates, which is consistent with what is commonly described world-wide [17, 22, 34]. The *mup*A gene is usually carried on plasmids, however a previous study identified a chromosomally encoded *mup*A gene in isolates with low-level resistance [35]. In our study neither *mup*A nor *mup*B were detected in the low-level resistant isolates. The *ile*S mutation V588F was the most common mechanism of low-level resistance, detected in 95% of low level resistant isolates, and is strongly linked to this phenotype [17]. In-vitro studies have shown that the presence of a single mutation (including the common *ile*S V588F) may have a small effect on bacterial growth rate. These mutations could occur after a single cycle of exposure to mupirocin, which provides selective pressure for low-level resistant MRSA strains within settings where mupirocin is commonly used [16, 17]. This could explain the high rate of low-level mupirocin resistance in our setting. A possibly novel *ileS* mutation, S570A, was identified in one of the low-level resistant isolates. However, further investigation is warranted since not all mutations within the *ile*S gene translate to phenotypic resistance [17], and only a small region of the *ile*S gene was sequenced in this study.

Certain strains were associated with mupirocin resistance; *spa* types t037 (linked to multilocus sequence type clonal complex (CC) 8) and t012 (linked to CC5) had strong associations with low-level resistance (mainly harbouring the mutation V588F), and t032 (linked to CC22) was associated with high-level (*mup*A) resistance. Studies within South Africa and even within Africa either did not correlate strain typing data with mupirocin resistance, or used typing methods which are not comparable across different laboratories [25, 36, 37]. In Africa, a single study from Ghana reported a mupirocin resistant isolate belonging to the *spa* type t4805 from a healthcare worker in Korle Bu Teaching Hospital [38]. In the United States, low-level resistance was predominantly reported amongst *spa* types linked to CC5 and CC8 [21, 39], consistent with the findings of our study. Furthermore, these genetic backgrounds (CC5 and CC8) are more prone to developing mutations within the *ile*S even following a single short treatment course with mupirocin [17]; which is consistent with the findings of this study.

## Conclusion

Although the method of selecting the *S. aureus* isolates from the biobank limited our ability to calculate the overall prevalence of mupirocin resistance, our data provided a baseline overview of the rates of resistance to mupirocin at Tygerberg Hospital. We reported high rates of low-level mupirocin resistance, driven by *spa* types t012 and t037, and low rates of high-level resistance, associated with *spa* type t032. Our study advocates for the continuous screening for mupirocin resistance in *S. aureus* from a wide range of clinical specimens in order to monitor resistance rates and to inform prescribing practices.

## Data Availability

Data sharing not applicable to this article as no datasets were generated or analysed during the current study.
